# The Effects of Hepatogomax Enteral Formula on Systemic Inflammation, Caecum Short-Chain Fatty Acid Levels, and Liver Histopathology in Thioacetamide-Induced Rats

**DOI:** 10.1155/2023/2313503

**Published:** 2023-09-14

**Authors:** Hery D. Purnomo, Refani A. Kusuma, Elfrida Sianturi, Ryan F. Haroen, Muchamad R. Solichin, Choirun Nissa, Adriyan Pramono, Endang Mahati, Etika R. Noer

**Affiliations:** ^1^Division of Gastroentero-Hepatology, Department of Internal Medical, Dr Kariadi Hospital, Faculty of Medicine, Diponegoro University, Semarang 50275, Indonesia; ^2^Department of Nutrition Science, Faculty of Medicine, Diponegoro University, Semarang 50275, Indonesia; ^3^Department of Anatomical Pathology, Faculty of Medicine, Public Health and Nursing, Gadjah Mada University, Yogyakarta 55281, Indonesia; ^4^Department of Pharmacology and Therapy, Faculty of Medicine, Diponegoro University, Semarang 50275, Indonesia

## Abstract

Liver damage characterized by fibrosis and necrosis can worsen the condition of liver disease. Liver disease is associated with impaired immune response and may affect short-chain fatty acid (SCFA) gut metabolites. Hepatogomax enteral formula was developed, which contains brain-chain amino acids (BCAAs) and middle-chain triglycerides (MCTs), which could repair liver tissue damage, improve the inflammatory status, and modulate SCFA in liver damage. The study aimed to determine the effect of hepatogomax on liver tissue repair, inflammation (TNF-*α* and IL-6), and SCFA levels in thioacetamide (TAA)-induced rats. The induction of TAA causes liver steatosis, increasing TNF-*α* and IL-6, and decreasing SCFA levels. Hepatogomax at a dose of 14.6 g/200 gBW significantly reduces TNF-*α* and IL-6 levels and increases SCFA levels (*p* < 0.05). The number of steatosis between groups P2 and P3 was lower as compared to a group of negative control [K2] (*p* < 0.05). Hepatogomax, in a dose-dependent manner, may repair liver tissue and improve inflammatory response and SCFA levels in TAA-induced rats.

## 1. Introduction

Liver is a vital organ that plays a role in human metabolism. Liver disease has become one of the major health problems worldwide, with mortality reaching 2 million per year [1, 2]. Liver disease can be caused by hepatotoxic agents that damage the liver's cell tissue. Thioacetamide (TAA) is one of the hepatotoxic chemical compounds used to induce fibrosis that resembles liver cirrhosis in humans in experimental studies [3]. Liver cirrhosis is liver disease's end stage, characterized by increased dead cells and induced liver fibrosis [4]. Hepatic fibrosis has been suggested as one of the hallmarks of nonalcoholic steatohepatitis (NASH), characterized by extensive accumulation of connective tissue following extensive tissue damage [5]. Of note, multiple cells play a role in the progression of liver disease, including hepatocytes, sinusoidal endothelial cells (SECs), and Kupffer cells (KCs) [6]. Furthermore, these cells are involved in cirrhosis development by releasing antigen-presenting cells for viruses, reactive oxygen species (ROS), and inflammatory mediators [7]. It has been shown that the increase in inflammatory response in the tissue of liver disease is associated with systemic inflammation [8].

Furthermore, it has been indicated that a hallmark of liver steatosis is characterized by the accumulation of bone marrow-derived macrophages and neutrophils of hepatocytes, Kupffer cells, and liver sinusoidal endothelial cells. This immune cell activation induces harmful inflammation and leads to NASH progression to cirrhosis [9]. Of interest, under NASH progression, there is a shift in the inflammatory state from the production of the anti-inflammatory cytokines to the production of the proinflammatory cytokines such as tumor necrosis factor-*α* (TNF-*α*) and interleukin-6 (IL-6), which play an important role in the liver regeneration process [10, 11]. More interestingly, recent studies have suggested a cross-talk between liver inflammation, systemic inflammation, and gut microbiota dysbiosis [12, 13]. The most recent human study demonstrated that an altered gut microbiome parallelly occurred during an acute-on-chronic liver failure (ACLF) stage. It has been determined that ACLF is the most severe clinical stage of cirrhosis [14].

Altered gut microbiome in liver disease may determine changes in the gut microbiota metabolites such as short-chain fatty acid (SCFA). It has been widely investigated that SCFA production is not only observed in the stool but also can bypass the systemic level (i.e., in the blood). A cross-sectional study in cirrhosis patients showed that low serum butyrate level was negatively associated with increased metabolic endotoxemia and systemic inflammation [15]. More interestingly, not only SCFA is decreased in cirrhosis but also another gut metabolite, brain-chain amino acid (BCAA), is decreased. The BCAA concentrations are low in the end stage of liver disease and may be associated with liver fibrosis, oxidative stress, and the development of a proinflammatory state in liver disease [16, 17]. BCAA administration in patients with liver disease can prevent progressive liver damage, stimulate protein and cell regeneration, and improve liver function and body immunity [10, 11]. BCAA can increase protein synthesis related to the immune system. An increased immune response requires the formation of new cells, antigens, immunoglobulins, cytokines, cytokines response, and acute-phase proteins. In addition, BCAA can increase the number of intrahepatic lymphocytes and stimulate natural killer (NK) activity and lectin-dependent cytotoxic activity in the liver [12]. Treatment for liver disease using a formula feeding containing the BCAAs may be beneficial in reducing tissue damage in the liver, decreasing systemic inflammation, and modulating the SCFA levels.

The present study uses the hepatogomax enteral formula, made from goat's milk flour and soybean flour for liver diseases. This formula was developed by Rahmadanti et al. [18] in 2020 and has met the European Society for Clinical Nutrition and Metabolism (ESPEN) guideline for the composition of the enteral diet for liver disease. We hypothesized that hepatogomax might reduce the progression of liver tissue damage, decrease inflammation (TNF-*α* and IL-6), and modulate the caecum SCFA level.

## 2. Materials and Methods

### 2.1. Hepatogomax Enteral Formula

The hepatogomax enteral formula was made at the Food Technology Science Laboratory, Diponegoro University, and was made from soybean flour “*Kusuka Ubiku*,” goat's milk flour “*Skygoat*,” virgin coconut oil (VCO) “*Al Alfiat*,” maltodextrin, and granulated sugar. The dry ingredients were mixed and stirred using a mixer for 3 minutes, and then, VCO oil was added to it and stirred for 2 minutes. All the ingredients were stirred manually and then stirred using a mixer for 8 minutes until they became homogeneous. The formula that has been homogenized is sifted to form a smoother formula. The formula is put into an airtight plastic container and stored at room temperature [18]. The hepatogomax enteral formula contains an energy density of 1.17 kkal/ml, low fat <27.33%, medium protein digestibility 53.44%, BCAAs 2.09%, and medium-chain triglycerides (MCTs) 29.26% [18–21].

### 2.2. Experimental Animals

This study was a true experimental pre-post test with a control group design conducted at the Experimental Animal Laboratory, Center for Food and Nutrition Studies, Gadjah Mada University, Yogyakarta. The subject used in this study was male adult Sprague–Dawley rats aged 8–12 weeks with a body weight of 180–250 g. The rats were then randomly assigned into six groups consisting of six rats (*n* = 6) for each group. All groups received 20 g/d/rats of AD-II Comfeed as standard feed and drank water freely. The K1 group is healthy rats as control negative while the K2 group is TAA-induced rats as control positive. The groups of K2, K3, P1, P2, and P3 were induced with TAA at 400 mg/kg BW for two weeks to make liver damage condition. The group of K3 was given a commercial formula at a dose of 4.32 g/200 gBW. In contrast, the groups of P1, P2, and P3 were given hepatogomax enteral formula at a dose of 4.97 g/200 gBW, 9.73 g/200 gBW, and 14.6 g/200 gBW for 28 days.

Blood sampling was carried out twice: (1) preintervention and (2) postintervention. All rats were fasted for 12 hours before taking blood through the retro-orbital plexus. TNF-*α* and Il-6 levels were determined using the ELISA methods. After the study was completed, the rats were terminated and taken out of the caecum for SCFA analysis. After that, the liver tissue was collected and immediately stored in formalin 10% for further histopathology analysis.

### 2.3. SCFA Analysis

The caecum of the rats taken was cleaned using phosphate-buffered saline or sodium chloride (NaCl). After that, the caecum was stored at a temperature (of −80°C). Prior to analysis, the caecum samples were homogenized using Tissue Lyser (Qiagen®) and were centrifuged at 14000 rpm for 15 minutes to collect the supernatant and added 25% metaphosphoric acid to maintain the pH of the buffer in a ratio of 4 : 1. The supernatant was centrifuged again at 14000 rpm for 30 minutes [22, 23]. The supernatant samples were analyzed using gas chromatography to obtain SCFA levels. SCFA levels were analyzed at the Food Technology and Agricultural Products Testing Laboratory, Gadjah Mada University, Yogyakarta.

### 2.4. Histopathological Examination

The resected liver organs were fixed using 10% neutral buffered formalin and were made into paraffin blocks. These blocks were then cut 3-4 micrometres. After that, staining was performed using hematoxylin-eosin (H&E). A microscope (Olympus CX33, Evident ®) with a magnification of 400 times was used to examine the liver structure histopathologically. Histopathological analysis was carried out at the Department of Anatomical Pathology, Gadjah Mada University, Yogyakarta.

### 2.5. Ethical Clearance

This study was reviewed and approved by the Health Research Ethics Commission of the Faculty of Medicine, Diponegoro University, with Ethical Clearance No. 17/EC/H/FK-UNDIP/II/2022.

### 2.6. Data Analysis

The data were analyzed using SPSS IBM 21. All results are expressed as mean ± standard deviation (SD). The normality of data was tested by the Shapiro–Wilk test. Data were analyzed using the paired t-test if it was normally distributed and the Wilcoxon test if the data were not normally distributed to determine the effects of pre- and postintervention. Differences between groups (K1, K2, K3, P1, P2, and P3) were analyzed using the one-way ANOVA parametric test followed by the post hoc LSD test for data with normal distribution. Data were analyzed using the Kruskal-Wallis test, followed by the Mann-Whitney test for non-normally distributed data. Value *p* < 0.05 was considered to be significant.

## 3. Results

### 3.1. Effect of Hepatogomax Enteral Formula on Body Weight

The results of the average body weight of all groups increased during the study (see Table 1). The K2, K3, P1, P2, and P3 groups had lower body weight after being induced by TAA. There was a significant increase in body weight in all groups after TAA induction (*p* < 0.05). The mean weight change before and after TAA induction showed that all groups experienced a significant increase in body weight (*p* = 0.003). In addition, only K1 group had a significant difference in the increase in all groups (*p* < 0.05). After the intervention, it showed a significant increase in body weight in all groups (*p* < 0.001). The P3 group experiences more weight gain than the K3 group after the intervention. There was a significant difference in the mean increase in weight change between groups after the intervention (*p* < 0.001). The P3 group did not have a significant difference from the K1 and K3 groups (*p* > 0.05).

### 3.2. Effect of Hepatogomax Enteral Formula on Inflammatory Biomarkers

There was a significant difference in inflammatory status levels in all groups after the intervention (*p* < 0.05). The TAA-induced groups had higher TNF-*α* and IL-6 serum levels than the K1 group. After the intervention, the P3 group had lower levels of TNF-*α* and IL-6 serum compared to the K3 group and almost the same as the K1 group. The changes in the mean levels of TNF-*α* and IL-6 serum showed significant differences between groups (*p* < 0.05). Changes in the mean decrease in TNF-*α* and IL-6 serum levels, which were greatest in the P3 group, are 13.49 pg/ml and 72.05 pg/ml. The groups of P1, P2, and P3, which were given hepatogomax intervention, had significant differences from all control groups (*p* < 0.05) (see Table 2).

### 3.3. Effect of Hepatogomax Enteral Formula on SCFA

The results of acetic acid, propionic acid, and butyric acid levels between groups had significant differences (*p* < 0.05). Groups K3, P1, P2, and P3 had significant differences in SCFA levels compared to groups K1 and K2 (*p* < 0.05). The P3 group had the highest levels of acetic acid, propionic acid, and butyric acid than the TAA-induced groups (see Table 3).

### 3.4. Histopathology

**Figure 1:**
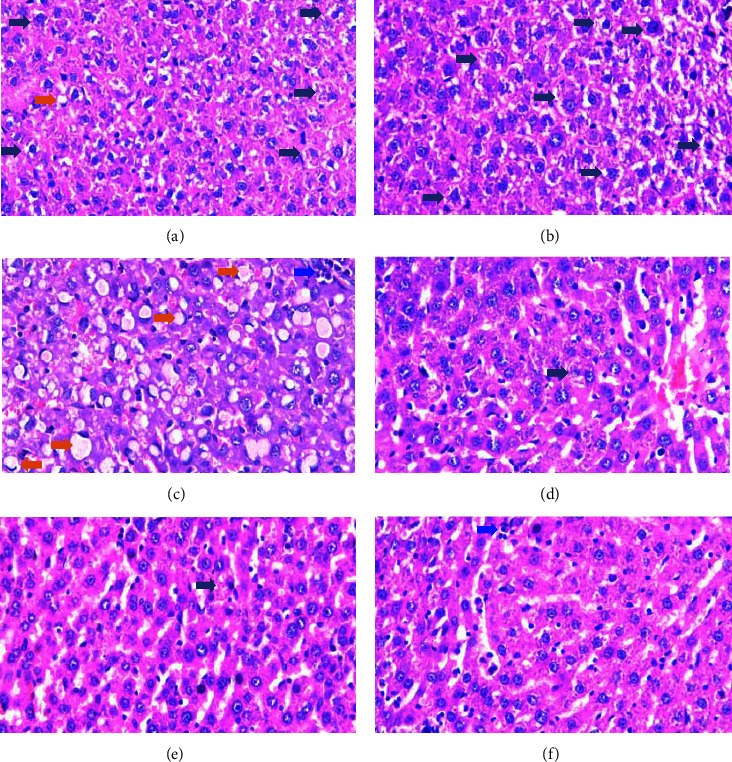
Histopathological liver. The orange arrow is steatosis, the navy arrow is ballooning cells, and the blue arrow is inflammatory necrosis. (a) Normal control; (b) TAA-induced liver cirrhosis control; (c) TAA + commercial formula 4.32 g/200 gBW; (d) TAA + hepatogomax 4.97 g/200 gBW; (e) TAA + hepatogomax 9.73 g/200 gBW; (f) TAA + hepatogomax 14.6 g/200 gBW (H&E; x400). The sections shown are from representative samples (*n* = 6/group).

Histopathological examination of the liver showed normal liver architecture with minimal inflammatory necrosis, ballooning cells, and steatosis (Figure 1(a)). TAA induction showed a severe increase in ballooning cells and large steatosis (Figure 1(b)). The administration of hepatogomax enteral formula at a dose of 4.97 g/200 gBW and 9.73 g/200 gBW showed a moderate ballooning cell and slight steatosis (Figures 1(d) and 1(e)). However, the administration of high doses of hepatogomax showed a slight increase in inflammatory necrosis and severe ballooning cells with barely noticeable steatosis (Figure 1(f)). The result of histopathological analysis showed no difference in the number of necrosis, ballooning, and inflammatory cells in all groups. However, there was a significant difference in the amount of steatosis (*p* < 0.05). Groups P1 and P2 significantly differed from group K2 (see Table 4).

## 4. Discussion

In this study, we demonstrated that hepatogomax formula feeding containing BCAAs and medium-chain triglycerides (MCTs) increases body weight, reduces systemic inflammations (markedly by a decrease in TNF-*α* and IL-6), modulates the caecum SCFA levels (acetate, butyrate, and propionate), and prevents progression of tissue damage in the liver of TAA-induced rats model. Thus, the results of this study suggest that hepatogomax formula feeding may influence the repairing of inflammation and tissue cell damage in the liver by modulating the production of SCFA by the gut microbiota associated with BCAAs.

Body weight before TAA-induced in all groups of rats was homogeneous and corresponded to the research inclusion criteria, 180–250 g. The results showed a lower increase in body weight before intervention in the K2, K3, P1, P2, and P3 groups compared to the K1 group. A lower increase in that group was due to TAA induction. This is due to a previous study showing that the group of rats induced by TAA 400 mg/kgBW for two weeks had a lower body weight (191.93 g) compared to the healthy rat group (244.86 g) [3]. TAA is a hepatotoxic compound that can cause centrilobular necrosis with a regenerative response of the liver that leads to the formation of liver cirrhosis [24]. Cirrhosis conditions in the liver cause a decrease in liver function, so malnutrition can occur, characterized by weight loss or low weight gain. Malnutrition in liver cirrhosis patients is caused by various factors, such as hypermetabolism due to infection, fat malabsorption, and nutritional metabolic disorders related to increased gluconeogenesis, protein catabolism, and decreased glycogenolysis [25].

The increase in body weight during intervention showed that giving hepatogomax formula enteral which contained macronutrients, 4.59 g of BCAA, and 76.66 g of MCT was able to repair damage to the liver so that there was an increase in body weight [21]. The condition of liver cirrhosis is related to insulin resistance, which causes the inability of the body to inhibit the gluconeogenesis process resulting in weight loss [26]. BCAA can improve insulin resistance in the liver by increasing sterol regulatory elements that bind to the protein-1c pathway, activating liver-type glucokinase (L-GK), and glucose transporters. Furthermore, BCAA suppresses liver expression of glucose-6-phosphatase (G6P) and increases peroxisome proliferator activator receptor (PPAR-*γ*) and uncoupling protein 2 (UCP2), which can stimulate free fatty acids oxidation [16, 27]. L-GK expression plays a role in reducing blood glucose by increasing glycogen synthesis or reducing gluconeogenesis which causes an increase in muscle mass and protein tissue to increase body weight [27–29]. Besides BCAA, MCT plays a role in improving muscle function and strength through the activation of protein kinase B (Akt) and signaling of the adenosine monophosphate protein kinase (AMPK) pathway, inhibiting, transforming growth factor-*β* (TGF-*β*) signaling, and increased metabolism in skeletal muscle [30].

Increased serum levels of TNF-*α* and IL-6 after TAA induction are associated with inflammation, proliferation, apoptosis, and fibrosis in the liver [31]. TAA is capable of causing liver damage by forming reactive oxidative metabolites, namely, S-oxide and SS-dioxide [32]. These result in fatty acid accumulation, protein and DNA damage, and the formation of reactive oxygen species (ROS), followed by the synthesis of a cascade of proinflammatory cytokines such as TNF-*α* and IL-6 [33]. Hepatogomax, which contains BCAAs and MCTs, can reduce TNF-*α* and IL-6 by decreasing ROS production by activating the antioxidant mechanism, thereby reducing oxidative stress and inflammation in the liver. Improvement of inflammation in the liver allows a decrease in TNF-*α* and IL-6 levels in liver damage conditions [34].

Patients with liver damage had a lower abundance of SCFA-producing bacterial species [35]. Decreased SCFA content can impair intestinal barrier function and increase circulating permeability. This study showed that hepatogomax formula feeding increased SCFA levels in TAA-induced rats with liver damage. The increase in SCFA levels was associated with a dose-dependent manner in which high doses of hepatogomax increased SCFA levels the most. The increase in SCFA levels indicated that the BCAAs and MCTs content in the hepatogomax enteral formula increased the composition of the bacteria-producing SCFA. BCAAs, through glycolysis, are converted into pyruvate, the precursor of three SCFAs. Gut microbiota such as *Anaerostipes, Faecalibaterium*, and *Clostridium* use pyruvate to produce SCFA acetate and butyrate via the acetyl-CoA pathway. Then, propionate is produced via the acetyl-CoA and succinate pathway by the gut microbiota *Coprococcus* and *Dialister* [36, 37].

This study demonstrated that elevated SCFA levels were inversely related to TAA-induced systemic inflammation. This is similar to LPS in liver inflammation, which is related to the activation of inflammasome nod-like receptor protein 3 (NLRP3) and the production of interleukin (IL)-1*β* through activation of the nuclear factor-*κ*B (NF-*κ*B) pathway [38]. NF-*κ*B is a key transcription factor of M1 macrophages. It is required to induce the number of inflammatory genes such as TNF-*α* and IL-6 [39]. SCFA can inhibit lipopolysaccharide (LPS)-induced inflammation [40]. SCFA reduces systemic inflammation by suppressing LPS production and maintaining intestinal integrity so that circulating LPS levels decrease. In addition, SCFA decreases the liver's inflammatory response by inhibiting the activity of histone acetyltransferases reducing the generation of regulatory T cells and the expression of cytokines in T cells. Propionate increased the inhibitory activity of Treg cells, while butyrate inhibited the activity of the NLRP3 inflammasome and increased the differentiation of regulatory T cells. Then, SCFA butyrate and propionate suppress NF-*κ*B activation resulting in a decrease in the production of proinflammatory cytokines [36, 41, 42]. Apart from that, based on other studies, it was explained that the production of various metabolites of the gut microbiota, such as SCFA (acetate, propionate, and butyrate), which is produced the most in the caecum, shows anti-inflammatory properties [43].

The content of MCTs and BCAAs can reduce the accumulation of fat in the liver by preventing fat malabsorption. Decreased fat malabsorption causes an increase in bile acid secretion into the intestinal lumen, which is related to an increased composition of SCFA-producing bacteria [44, 45]. BCAAs also contribute to energy homeostasis and lipid metabolism and have an anti-inflammatory effect that can suppress inflammatory reactions in the intestine, so that gut microbiota of SCFA production increases [45]. Also, BCAAs are precursors of microbial-derived SCFA in the gut. It causes an increase in the bioavailability of SCFA [46]. Of interest, it has been suggested that the BCAAs from exogen (i.e., from the diet) may shape the gut microbiota *Ruminococcus favefaciens*. This leads to an increase in the bioavailability of acetate, where acetate will decrease the expression of lipogenesis genes, resulting in reduced fat accumulation and steatosis in the liver [45].

In addition, lower necrosis and ballooning cells in the P1 and P2 groups suggest that BCAAs-enabled repair of liver tissue damage is possible. BCAAs could attenuate liver inflammation, including IL-6, by suppressing the activation of the LPS-binding protein, toll-like receptor 4, and signal transduction and activator of transcription-3 (LBP-TLR4-STAT3) pathway and reducing the translocation microbiota of *Enterococcus faecalis* into the liver. Suppressing LBP expression and STAT3 activation reduced hepatocyte death in liver damage conditions [47]. However, this study showed severe ballooning cells in the P3 group. There may be an unfavorable effect on the tissues when given high doses.

This study has certain limitations. First, we did not perform an analysis of the gut microbiome, serum LBP or LPS levels, other inflammation markers, and markers related to liver damage and steatosis, which could benefit from observing the underlying mechanism. Furthermore, we did not analyze serum BCAA levels to determine whether the improvement in all variables was based on the increase in serum BCAAs. Parameters in our study are used for hypotheses and mechanism bridges, but further research is needed with other parameters to be able to explain the improvement of the pathophysiological mechanism of the effect of the hepatogomax formula. Finally, we need further research on the mechanism of side effects of high-dose BCAAs and MCTs in the hepatogomax enteral formula against damaged liver tissue.

## 5. Conclusions

The present study showed that hepatogomax enteral formula administration on TAA-induced may improve systemic inflammation, increase SCFA levels, and reduce steatosis in a dose-dependent manner. Then, we need further research about the mechanism involved.

## Figures and Tables

**Table 1:** Body weight of rats.

Body weight (g)	Means ± SD				*p* ^1^	*p* ^2^	Means ± SD	
	Initial	Before TAA-induced	Before intervention	After intervention			ΔBB1	ΔBB2
**K1**	182 ± 3.46	189.17 ± 2.86	203.5 ± 2.43	232.5 ± 3.51	<0.001^1*∗*^	<0.001^1*∗*^	14.33 ± 1.03^a,b^	29 ± 1.67^a,b^
**K2**	179.5 ± 4.59	186.17 ± 4.36	191.83 ± 4.79	201 ± 6.1	<0.001^1*∗*^	<0.001^1*∗*^	5.67 ± 0.82^a^	9.17 ± 1.72^a,b^
**K3**	180.33 ± 2.94	187.67 ± 2.58	194.83 ± 3.19	223.83 ± 3.49	<0.001^1*∗*^	<0.001^1*∗*^	7.17 ± 2.04^a^	29 ± 1.79^a^
**P1**	181 ± 3.35	188.5 ± 3.02	194.67 ± 3.78	211.83 ± 4.96	<0.001^1*∗*^	<0.001^1*∗*^	6.17 ± 0.98^a^	17.17 ± 1.83^a,b^
**P2**	178.17 ± 3.31	185.33 ± 3.72	191.67 ± 3.93	217.67 ± 4.32	<0.001^1*∗*^	<0.001^1*∗*^	6.33 ± 0.82^a^	26 ± 1.10^a,b^
**P3**	179.5 ± 3.78	186.67 ± 3.44	192.67 ± 3.61	221.17 ± 3.82	<0.001^1*∗*^	<0.001^1*∗*^	6.00 ± 0.63^a^	28.5 ± 0.55^b^
*p* ^3^							0.003^2*∗*^	<0.001^2*∗*^

^1^Paired *t*-test, *p*^1^ = *p* value before TAA-induced and before intervention; *p*^2^ = *pvalue* before and after intervention; ^*∗*^significant difference (*p* < 0.05); ΔBB1 = BW change before TAA-induced and before intervention; ΔBB2 = BW before and after intervention; ^2^*Kruskal–Wallis* test; ^*∗*^significant difference (*p* < 0.05); ^a^significant difference against the control group (*Mann–Whitney* test); ^b^significant difference against the intervention group (*Mann–Whitney* test).

**Table 2:** Inflammatory biomarker before and after intervention.

Variable	Preintervention	Postintervention	*p*	Δ Changes	*p*
	Mean ± SD	Mean ± SD		Mean ± SD	
TNF-*α* (pg/ml)					<0.001^3*∗*^
K1	6.62 ± 0.21	7.01 ± 0.32	0.027^1*∗*^	0.38 ± 0.20^b^	
K2	20.70 ± 0.26	20.97 ± 0.29	0.006^2*∗*^	0.27 ± 0.14^b^	
K3	20.88 ± 0.25	7.89 ± 0.29	<0.001^2*∗*^	−12.99 ± 0.42^a,b^	
P1	20.91 ± 0.20	15.06 ± 0.34	0.028^2*∗*^	−5.85 ± 0.35^a,b^	
P2	20.85 ± 0.20	9.33 ± 0.38	<0.001^2*∗*^	−11.47 ± 0.45^a,b^	
P3	20.78 ± 0.16	7.30 ± 0.21	<0.001^2*∗*^	−13.49 ± 0.28^a,b^	
IL-6 (pg/ml)					<0.001^4*∗*^
K1	66.5 ± 2.47	70.82 ± 2.87	0.002^2*∗*^	4.32 ± 1.86^d^	
K2	148.67 ± 5.30	153.153 ± 5.39	0.008^2*∗*^	4.49 ± 2.57^d^	
K3	151.16 ± 5.61	83.77 ± 3.43	<0.001^2*∗*^	−67.40 ± 3.81^c,d^	
P1	152.32 ± 5.03	117.63 ± 4.17	<0.001^2*∗*^	−34.70 ± 4.60^c,d^	
P2	150.17 ± 4.15	92.23 ± 3.21	<0.001^2*∗*^	−57.93 ± 5.79^c,d^	
P3	151.16 ± 7.17	79.17 ± 4.69	<0.001^2*∗*^	−72.05 ± 7.55^c,d^	

^1^Wilcoxon test, ^2^paired *t*-test, ^3^Kruskal–Wallis test, ^4^one-way ANOVA test, *p* = *p* value, ^*∗*^significant difference (*p* < 0.05), ^a^significant difference against control group (*Mann*–*Whitney* test), ^b^significant difference against intervention group (*Mann*–*Whitney* test), ^c^significant difference against control group (*post hoc LSD* test), ^d^significant difference against intervention group (*post hoc LSD* test).

**Table 3:** SCFA levels after intervention.

SCFA (m Mol)	Mean ± SD						*p* ^1^
	K1	K2	K3	P1	P2	P3	
Acetic acid	4.29 ± 0.77^a,b^	1.85 ± 0.48^a,b^	2.11 ± 0.77^a,b^	2.20 ± 0.46^a,b^	2.18 ± 0.75^a,b^	3.13 ± 0.97^a,b^	<0.001^*∗*^
Propionic acid	3.09 ± 0.60^a,b^	1.54 ± 0.33^a,b^	1.75 ± 0.58^a,b^	1.81 ± 0.43^a,b^	1.84 ± 0.62^a,b^	2.54 ± 0.68^a,b^	<0.001^*∗*^
Butyric acid	1.46 ± 0.27^a,b^	0.78 ± 0.19^a,b^	0.90 ± 0.29^a,b^	0.92 ± 0.20^a,b^	0.95 ± 0.25^a^	1.25 ± 0.32^a,b^	0.001^*∗*^

^1^One-way ANOVA test; *p* = *p* value; ^*∗*^significant difference (*p* < 0.05); ^a^significant difference against control group (post hoc LSD test); ^b^significant difference against intervention group (post hoc LSD test).

**Table 4:** Histopathology liver analysis.

Histopathology liver	Mean ± SD			
	Necrosis (%)	Steatosis (%)	Ballooning cells (%)	Inflammatory cells (%)
K1	1.17 ± 1.94	4.50 ± 4.60^a^	35.00 ± 33.46	1.00 ± 0.63
K2	0.50 ± 0.55	15.17 ± 10.21^a,b^	62.5 ± 25.45	0.67 ± 0.52
K3	0.17 ± 0.41	11.00 ± 10.95^b^	46.67 ± 31.25	0.67 ± 0.52
P1	0.00 ± 0.00	5.33 ± 12.09	30.17 ± 28.43	0.50 ± 0.55
P2	0.67 ± 0.52	1.00 ± 2.00^a^	27.83 ± 28.91	1.00 ± 0.00
P3	0.50 ± 0.55	1.33 ± 1.86^a^	50.83 ± 31.37	1.00 ± 0.00
*p* ^1^	0.179	0.021^*∗*^	0.290	0.236

^1^Kruskal–Wallis test; *p* = *p* value; ^*∗*^significant difference (*p* < 0.05); ^a^significant difference against the control group (Mann–Whitney test); ^b^significant difference against the intervention group (Mann–Whitney test).

## Data Availability

The data are available upon request to the corresponding author via e-mail.
